# Subjective responses to emotional body odors and common odors in autism-spectrum disorders

**DOI:** 10.1192/j.eurpsy.2021.1630

**Published:** 2021-08-13

**Authors:** J. Grave, J. Noll, F. Barros, L. Kogler, J. Freiherr, D. Wildgruber, S. Soares, B. Derntl

**Affiliations:** 1 William James Center For Research, Department Of Education And Psychology, University of Aveiro, Aveiro, Portugal; 2 Centre For Research In Health Technologies And Services, Department Of Education And Psychology, University of Aveiro, Aveiro, Portugal; 3 Faculty Of Medicine, Department Of Psychiatry And Psychotherapy, University of Tübingen, Tübingen, Germany; 4 Department Of Sensory Analytics, Fraunhofer Institute for Process Engineering and Packaging (IVV), Freising, Germany; 5 Lead Graduate School And Research Network, University of Tübingen, Tübingen, Germany; 6 Werner Reichardt Centre For Integrative Neuroscience, University of Tübingen, Tübingen, Germany

**Keywords:** Autism-spectrum disorders, olfaction, body odor, emotion

## Abstract

**Introduction:**

Autism-spectrum disorders (ASD) are characterized by deficits in social domains, associated with abnormal socioemotional perception. Although olfaction provides access to socioemotional cues, little is known about the perception of emotional odors considering their social meaning in ASD.

**Objectives:**

To investigate the subjective responses to emotional body odors (BOs) versus non-social, common odors (COs) in ASD.

**Methods:**

Eleven ASD and 49 typically developed (TD) adults were asked to smell negative, positive, and neutral BOs (axillary sweat from healthy individuals exposed to fearful, happy, and neutral film-clips) and COs, and to rate each odor on perceived pleasantness, intensity, familiarity and arousal. Odors were presented for 5 sec. Analyses were performed with linear mixed-effect models with fixed factors (group × odor type × valence) and covariates (e.g., age; intensity for arousal/familiarity; familiarity for pleasantness). Post-hoc comparisons were Bonferroni-corrected.

**Results:**

Odors were perceived as significantly more intense (p=.044) and pleasant (p<.001) in ASD than TD. Distinct response patterns were found in ASD and TD. First, positive BOs and COs were similarly arousing and pleasant in ASD (p>.05), but not in TD (p<.001). Second, positive and neutral COs were equally arousing, familiar and pleasant in ASD (p>.05), but not in TD (p<.001). No differences were observed between BOs in ASD and TD (p>.05).

**Conclusions:**

ASD is associated with abnormal subjective responses to emotional odors, which could contribute to the social communication difficulties characterizing ASD. Since emotional BOs elicit psychological responses in others, analyses on subjective and automatic responses will allow a better understanding of the role of olfaction in ASD.

**Disclosure:**

No significant relationships.

